# Cryptotanshinone from *Salvia miltiorrhiza* Bunge (Danshen) inhibited inflammatory responses via TLR4/MyD88 signaling pathway

**DOI:** 10.1186/s13020-020-00303-3

**Published:** 2020-03-02

**Authors:** Xin-Xing Li, Xiaoting Zheng, Zhenjie Liu, Qiongming Xu, Hongzhen Tang, Jianfang Feng, Shilin Yang, Chi Teng Vong, Hongwei Gao, Yitao Wang

**Affiliations:** 1 College of Pharmacy, Guangxi University of Chinese Medicine, Nanning, 530000 China; 2 Guangxi Engineering Technology Research Center of Advantage Chinese Patent Drug and Ethnic Drug Development, Nanning, 530020 China; 3 State Key Laboratory of Quality Research in Chinese Medicine, Institute of Chinese Medical Sciences, University of Macau, Room 1050, N22 Research Building, Macao, China; 4 College of Pharmaceutical Science, Soochow University, Suzhou, 215123 China

**Keywords:** Cryptotanshinone, Anti-inflammation, TLR4-MyD88, PI3K/AKT, Nrf2

## Abstract

**Background:**

Cryptotanshinone (CPT), as a major component of *Salvia miltiorrhiza* Bunge (Danshen), displays many pharmacological activities including anti-inflammatory effects. However, the exact cellular and molecular mechanisms of the anti-inflammatory activities of CPT remain to be elucidated. The present study was aimed to clarify its mechanisms on lipopolysaccharide (LPS)-induced inflammatory responses in mouse macrophages, RAW264.7 cells.

**Methods:**

In the current study, the anti-inflammatory properties of CPT were evaluated using LPS-stimulated RAW264.7 cell model. MTT assay was used to determine the viability of RAW264.7 cells. The anti-inflammatory effects of CPT were measured based on the detection of nitric oxide (NO) production (Griess and flow cytometry assay), and tumor necrosis factor-α (TNF-α) and interleukin-6 (IL-6) release (ELISA). Cyclooxygenase-2 (COX-2) and inducible nitric oxide synthase (iNOS) enzyme expressions were also determined by western blotting. Besides, by using flow cytometry, we also evaluated the effect of CPT on LPS-induced calcium influx. Finally, the underlying anti-inflammatory mechanisms of CPT were investigated using western blotting to assess the protein levels of toll-like receptor 4 (TLR4), myeloid differentiation factor 88 (MyD88), phosphatidylinositol 3-kinase (PI3K)/AKT, nuclear factor erythroid 2 related factor 2 (Nrf2), mitogen-activated protein kinase (MAPK), and nuclear factor-kappa B (NF-κB) pathways.

**Results:**

Our data showed that CPT inhibited LPS-induced pro-inflammatory cytokine release like IL-6, and TNF-α, as well as NO production. It displayed a significant inhibitory effect on the protein expressions such as iNOS, COX-2, NF-κB pathway like inhibitor of kappa B kinase (IKK)α/β, inhibitor of kappa B (IκB)-α and NF-κB/p65, PI3K/AKT pathway like PI3K and AKT, and MAPK pathway like c-Jun N-terminal kinase (JNK)1/2, extracellular signal-regulated kinase (ERK)1/2, and p38, in LPS-stimulated RAW264.7 macrophages. Moreover, the immunofluorescence results indicated that CPT suppressed NF-κB/p65 translocation from the cytoplasm into the nucleus. Further investigations showed that CPT treatment increased NAD(P)H quinone oxidoreductase-1 (NQO1) and heme oxygenase-1 (HO-1) expressions together with its upstream mediator, Nrf2. In addition, CPT inhibited LPS-induced toll-like receptor 4 (TLR4) and MyD88 expressions in RAW264.7 macrophages.

**Conclusions:**

Collectively, we suggested that CPT exerted significant anti-inflammatory effects via modulating TLR4-MyD88/PI3K/Nrf2 and TLR4-MyD88/NF-κB/MAPK pathways.

## Background

Inflammation plays a pivotal role in the pathophysiology of atherosclerosis, metabolic disorders, ulcerative colitis, diabetes, and obesity [1]. It is beneficial for the treatment of inflammatory-related diseases to clearly understand the mechanisms of inflammation [2, 3]. Toll-like receptor-4 (TLR4), one of the best characterized pattern recognition receptors, is highly related to inflammatory responses [4]. It forms a heterodimer with MD-2 that recognizes lipopolysaccharide (LPS) [5]. LPS, a member of stimuli, can activate macrophages by binding to its receptor, TLR4, which activates the intracellular signaling cascade by recruiting myeloid differentiation factor 88 (MyD88) to the membrane, and ultimately induces the translocation of nuclear factor-kappa B (NF-κB) and pro-inflammatory responses [6, 7]. Upon stimulation by LPS, activation of NF-κB can regulate the gene transcription of the corresponding target genes [8]. NF-κB, bound by its endogenous inhibitor, inhibitor of kappa B α (IκBα), is an inactive complex in the cytoplasm of the resting cells. When stimulated, the complex between NF-κB and IκBα is dissociated, and then the dissociated NF-κB enters the cell nucleus, which regulates the transcription of pro-inflammatory cytokines and inducible enzymes such as tumor necrosis factor (TNF)-α, interleukin (IL)-6, and IL-1β, and cyclooxygenase-2 (COX-2) and inducible nitric oxide synthase (iNOS) [9]. In addition, the activation of phosphatidylinositol 3-kinase (PI3K)/AKT plays an important role in the expression of iNOS and COX-2 in mesangial cells and peritoneal macrophages [10]. PI3K activation leads to the phosphorylation of phosphatidylinositides, which then activates the downstream target, AKT, and activates the inflammatory responses [11]. Mitogen-activated protein kinase (MAPK), another momentous signaling pathway, possesses a prominent role in inflammatory processes [12]. The activation of MAPK pathway can be indicated by the up-regulation of p38, extracellular signal-regulated kinase (ERK), and c-Jun N-terminal kinase (JNK) [13].

In macrophages, calcium, as a second signal messenger, plays an important role in the activation of macrophages, such as transcriptional control, and the activation of kinases, and phosphatases [4, 14]. An increase in intracellular Ca^2+^ in accordance with LPS stimulation has been shown to be involved in the inflammatory process for the transcriptional activation, and leads to TNF-α, IL-6, IL-1β, and NO release [4, 14].

Previous study demonstrated that oxidative stress is always observed during the inflammatory processes [15]. Nuclear factor erythroid 2 related factor 2 (Nrf2) signaling pathway plays a significant role in protecting cells from oxidative stress. Under quiescent conditions, the transcription factor, Nrf2, interacts with the actin-anchored protein, Kelch-like ECH-associated protein 1 (Keap-1), which is largely localized in the cytoplasm. However, upon recognition of stimuli signals, Nrf2 is released from Keap-1, which escapes from proteasomal degradation, translocates to the nucleus, and transactivates the expressions of several cytoprotective genes, including NAD(P)H: quinone oxidoreductase-1 (NQO1) and heme oxygenase-1 (HO-1) [16, 17]. Therefore, the enhancement of Nrf2 signaling could ameliorate inflammation.

*Salvia miltiorrhiza* Bunge (Danshen), a common traditional Chinese medicine, comprises lipid-soluble tanshinone, as well as water-soluble salvianolic acid and lithospermic acid [18]. Accumulating evidences indicated that tanshinone exhibited anti-inflammatory [19, 20], anti-cancer [21], and immunomodulatory effects [22]. Cryptotanshinone (CPT), a quality-control ingredient in Danshen, exhibits a diversity of bio-activities, especially anti-inflammation. Although previous studies indicated that CPT exhibited anti-inflammatory effects [23, 24], however the anti-inflammatory mechanisms of CPT have not been clearly understood. In the present study, by using LPS-stimulated RAW264.7 cells, we unraveled the detailed anti-inflammatory mechanisms of CPT in mouse macrophages.

## Materials and methods

### Chemicals and reagents

**Fig. 1:**
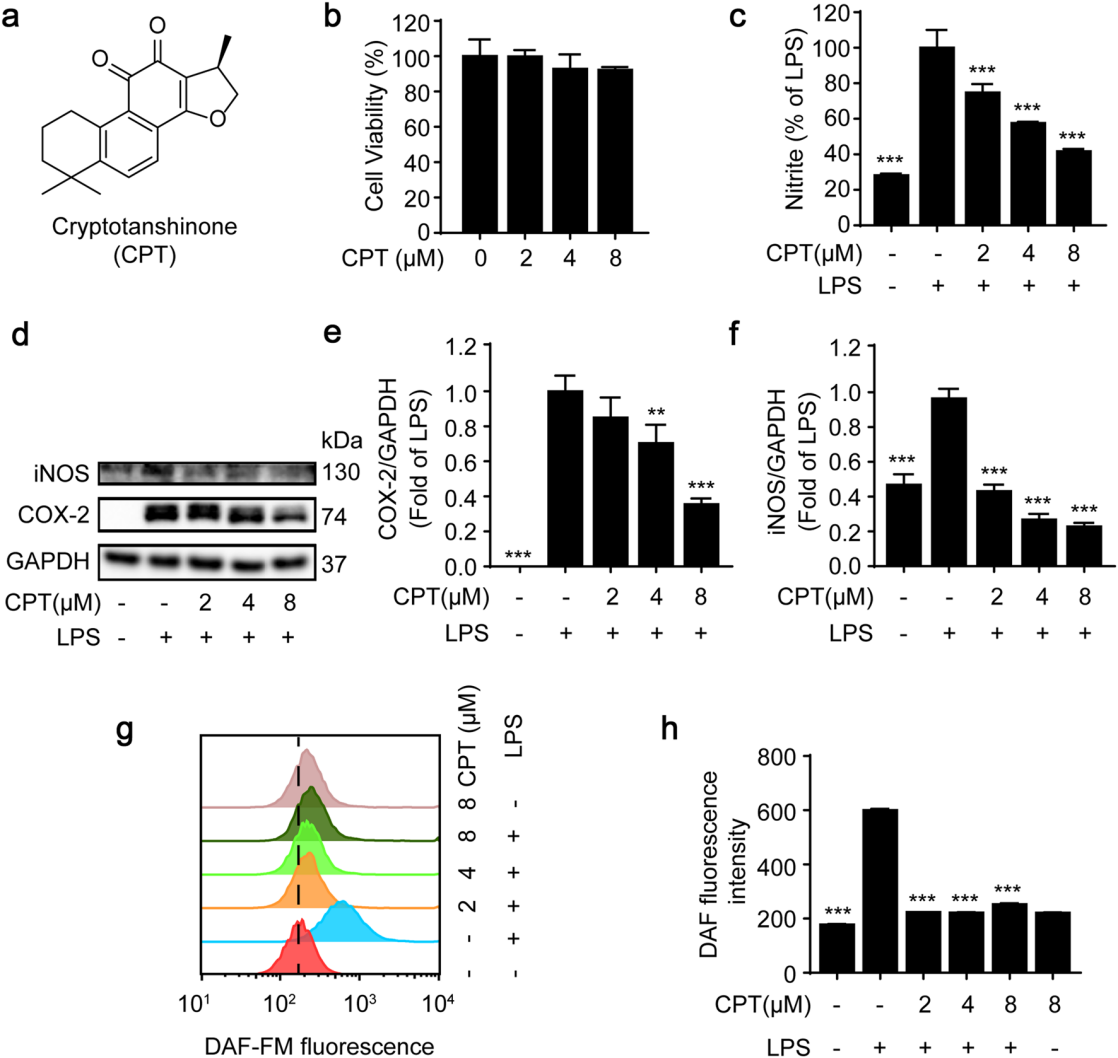
CPT induced anti-inflammatory activity in lipopolysaccharide (LPS)-stimulated RAW264.7 cells. **a** Chemical structure of CPT. **b** The cells were treated with CPT (0, 2, 4, 8 μM) for 24 h. The cell viability of CPT was determined by MTT assay. n = 4. **c** The cells were pretreated with CPT at indicated concentrations for 1 h before being stimulated with LPS (1 μg/mL) for another 24 h. The nitrite level was determined using Griess reagent assay. n = 4. **d**–**f** The cells were pretreated with CPT (0, 2, 4, 8 μM) for 1 h before stimulated with LPS (1 μg/mL) for another 24 h. The protein expressions of inducible nitric oxide synthase (iNOS) and cyclooxygenase-2 (COX-2) were determined by immunoblotting. n = 3. **g**–**h** The cells were pretreated with indicated concentrations of CPT (0, 2, 4, 8 μM) for 1 h before stimulated with LPS (1 μg/mL) for another 6 h. The nitric oxide (NO) level was determined by flow cytometry using DAF-FM staining (1 μM). n = 3. **p < 0.01, ***p < 0.001 compared with LPS alone group

CPT (C_19_H_20_O_3_, Fig. 1a), purity > 98%, was purchased from Chengdu Pufei De Biotech Co., Ltd (Chengdu, China), of which the certificate was shown in the supplementary information. LPS (*Escherichia coli* O111:B4), 3-(4, 5-dimethylthiazol-2-yl)-2,5-diphenyl tetrazolium bromide (MTT), Griess reagent (modified-G4410), dimethyl sulfoxide (DMSO), DAF-FM (NO detector), 4ʹ,6-Diamidino-2-phenylindole (DAPI), and Fluo-3/AM (Ca^2+^ detector) were obtained from Sigma-Aldrich (Louis, MO, USA). Fetal bovine serum (FBS), Dulbecco’s modified eagle medium (DMEM), penicillin and streptomycin were purchased from Gibco (Grand Island, NY, USA). The enzyme-linked immunosorbent assay (ELISA) kits for TNF-α and IL-6 were from Neobioscience (Shenzhen, China). BCA protein assay kit was purchased from Thermo Scientific (Waltham, MA, USA). The antibodies for GAPDH (#5174, 1:1000), COX-2 (#12282, 1:1000), iNOS (#39898, 1:1000), p-PI3K (#4228, 1:1000), PI3K (#4249, 1:1000), p-Akt (#4060, 1:2000), Akt (#4691, 1:1000), p-transforming growth factor beta-activated kinase 1 (Tak1) (#9339, 1:1000), Tak1 (#5206, 1:1000), p-ERK1/2 (#4370T, 1:2000), ERK1/2 (#4695T, 1:1000), p-JNK1/2 (#4668T, 1:1000), JNK1/2 (#9252T, 1:1000), p-p38 MAPK (#4511, 1:1000), p38 MAPK (#9212, 1:1000), p-IκBα (#2859, 1:1000), IκBα (#4814, 1:1000), p-inhibitor of kappa B kinase (IKK)α/β (#2078, 1:1000), IKKα (#2682, 1:1000), IKKβ (#8943, 1:1000), NF-κB/p65 (#8242T, 1:1000), p-p65 (#3033, 1:1000), Keap-1 (#4678, 1:1000), Nrf2 (#12721, 1:1000), HO-1 (#70081, 1:1000), and secondary antibodies were obtained from Cell Signaling Technology (Beverly, MA, USA). Antibodies against MD-2 (ab24182, 1 μg/mL), NQO1 (ab80588,1:10,000), MyD88 (ab199247, 1:10,000), and TLR4 (ab13867, 1 μg/mL) antibody were purchased from Abcam (Cambridge, MS, United States).

### Cell culture

RAW264.7 cells were purchased from the Shanghai Cell Bank of the Chinese Academy of Sciences (Shanghai, China). The cells were cultured in DMEM containing 10% FBS, 100 U/mL penicillin, and 100 U/mL streptomycin. These cells were maintained in a humidified incubator at 37 °C, where CO_2_ accounted for 5%.

### Cell viability assay

RAW264.7 cells were seeded onto 96-well plates at a density of 5 × 10^4^ cells per well overnight. Then the cells were exposed to various concentrations of CPT (0, 2, 4, 8 μM) for 24 h. MTT solution (5 mg/mL) were added for another 4 h. The supernatant in each well were removed and 100 μL of DMSO was added into the plate. The absorbance was measured at 570 nm wavelength with a microplate reader (BioTek, USA).

### Determination of nitrite in LPS-stimulated RAW264.7 cells

RAW264.7 cells (5 × 10^4^ cells per well) were seeded onto 96-well plates and cultured overnight. The cells were treated with CPT (0, 2, 4, 8 μM) for 1 h and then stimulated with LPS (1 μg/mL) for 18 h. The supernatant was collected to determine the nitrite levels.

### Mediator measurements by ELISA

According to our previous study [25], RAW264.7 cells were seeded onto 24-well plates at a density of 2 × 10^5^ cells/well overnight, and then the cells were treated with CPT (0, 2, 4, 8 μM) and stimulated with or without LPS (1 μg/mL) for 18 h. The supernatant was collected, and IL-6 and TNF-α levels were determined by ELISA kits according to the manufacturer’s instructions.

### Flow cytometry assay for the measurement of nitric oxide (NO) and calcium levels

The flow cytometric analyses of the measurement of NO and calcium levels were performed as previously described [4]. Briefly, 2 × 10^5^ RAW264.7 cells were seeded onto 24-well plates and cultured overnight. The cells were pretreated with CPT for 1 h followed by stimulation with LPS (1 μg/mL) for another 6 h. Then the cells were labeled with Fluo-3/AM (1 μM) and DAF-FM (1 μM) for further 1 h at 37 °C, respectively. Fluo-3/AM was used to measure calcium levels, while DAF-FM was used to determine NO production. Subsequently, the labeled cells were collected to measure the fluorescence intensities by a flow cytometer (BD LSRFortessa™ Cell Analyzer, BD, USA).

### Western blotting

Western Blotting was performed according to our previous study [21]. Briefly, the protein samples were collected from different CPT treatment groups (0, 2, 4, 8 μM) in the presence of LPS (1 μg/mL). The protein concentrations of the samples were determined with the BCA protein assay kit. The equal amount of proteins from each group were loaded onto SDS polyacrylamide gel electrophoresis (SDS-PAGE) gel to separate the target protein, and then the gels were electrophoretically transferred onto the polyvinylidene fluoride (PVDF) membranes. The membranes were blocked with 5% skimmed milk for 1 h at room temperature, then incubated with indicated primary antibodies overnight at 4 °C. The horseradish peroxidase (HRP) conjugated secondary antibody was then added and incubated for 2 h at room temperature. Finally, the bands of the target proteins were photographed by ChemiDoc™ MP Imaging System (Bio-Rad, CA, USA).

### Immunofluorescence and confocal images assay

RAW264.7 cells (2 × 10^5^ cells/dish) were seeded onto the confocal dish, and pretreated with or without CPT (8 μM) for 1 h, then the cells were treated with or without LPS (1 μg/mL) for 2 h. 4% paraformaldehyde was used to fix the cells for 30 min at room temperature. After that, Triton X-100 (0.1%) was used to permeabilize the cells, and the cells were subsequently incubated with NF-κB/p65 antibody overnight at 4 °C. After incubation, fluorochrome-conjugated secondary antibody was utilized to stained the cells for 1 h. The images were taken by a fluorescence microscope (Leica TCS SP5 laser confocal microscope, Leica Corporation, Wetzlar, Germany).

### Statistical analysis

Data were expressed as mean ± standard deviation (SD). GraphPad Prism 6.0 Software (GraphPad Software, San Diego, USA) was used to determine the statistically significant differences. One-way analysis of variance (ANOVA) followed by Dunnett’s post hoc test or two-way ANOVA followed by Bonferroni post hoc test was used to compare differences in more than two groups. *p *< 0.05 was considered as statistically significant.

## Results

### CPT exhibited anti-inflammatory properties in LPS-stimulated RAW264.7 macrophages

Increasing evidences suggested that inflammation is implicated in the development of a number of diseases, such as periodontal diseases, cardiovascular diseases, cancer, diabetics, rheumatoid arthritis, stroke, and aging [26, 27], which seriously threaten human health. In this study, the anti-inflammatory effects of CPT and the related mechanisms were investigated in mouse macrophages, RAW264.7 cells. The results showed that CPT (0–8 μM) showed no significant cytotoxicity (Fig. 1b). As shown in Fig. 1c, LPS induced a dramatic rise in nitrite levels in RAW264.7 cells, which could be reversed by CPT. In addition, we found that CPT inhibited LPS-induced up-regulation of iNOS and COX-2 in a concentration-dependent manner (Fig. 1d–f). Furthermore, the NO level was determined by flow cytometry. As shown in Fig. 1g, h, LPS induced a sharp increase of NO production, which was significantly inhibited by CPT pretreatment. Collectively, these results suggested that CPT displayed anti-inflammatory activities in RAW264.7 macrophages.

### CPT inhibited LPS-induced IL-6 and TNF-α release and calcium influx in RAW264.7 macrophages

**Fig. 2:**
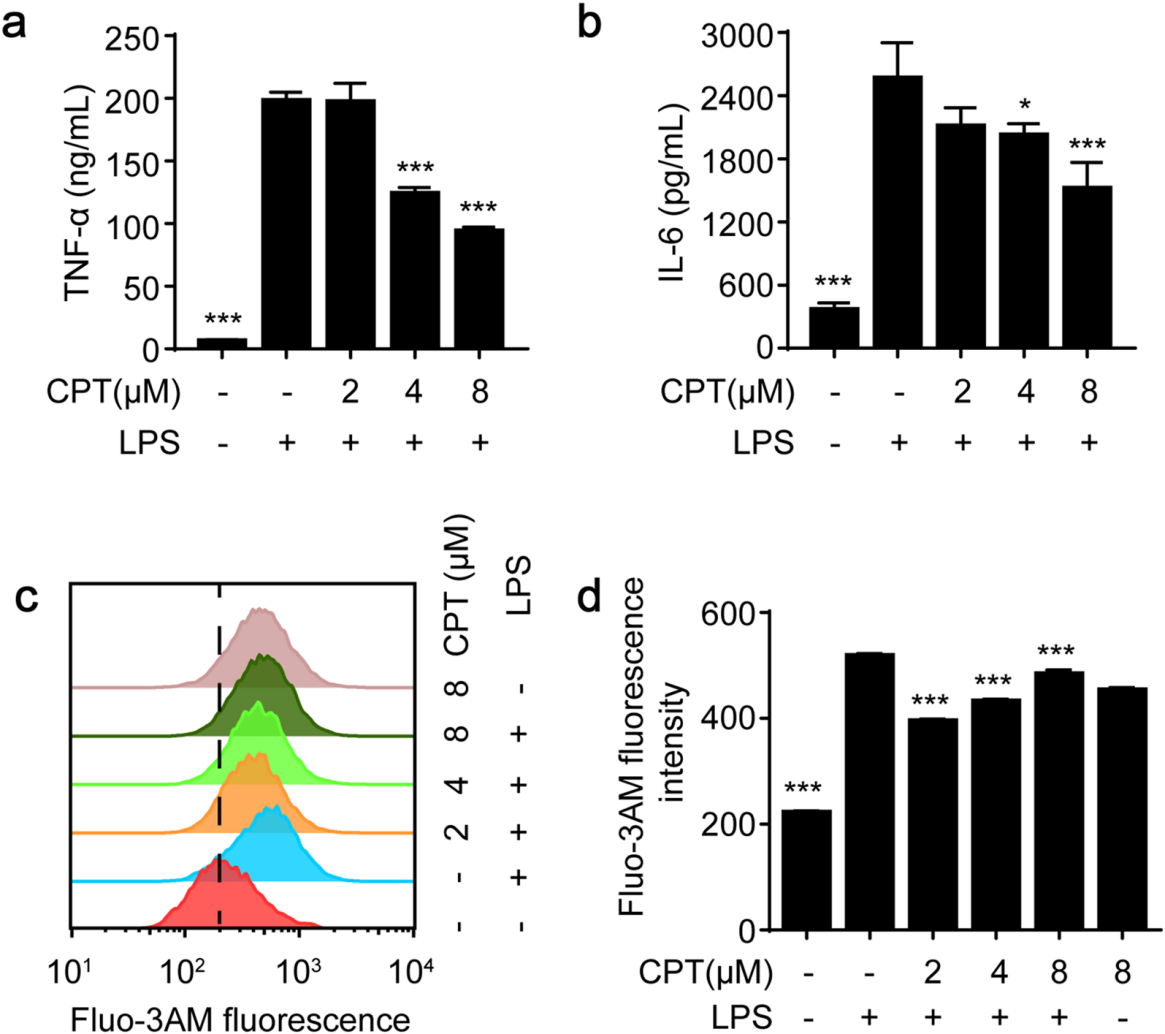
CPT inhibited TNF-α and IL-6 release as well as calcium influx in lipopolysaccharide (LPS)-stimulated RAW264.7 cells. **a**, **b** The cells were pretreated with CPT (0, 2, 4, 8 μM) for 1 h before being stimulated with LPS for another 24 h. The pro-inflammatory cytokines, TNF-α and IL-6, were determined by ELISA. n = 3. **c**, **d** The intracellular calcium levels were measured by flow cytometry with Fluo-3AM (1 μM). n = 3. *p < 0.05, ***p < 0.001 compared with LPS alone group

Inflammation is a complicated process which is regulated by a series of inflammatory mediators and cytokines [1]. Accumulating evidences have confirmed that pro-inflammatory cytokines, such as TNF-α and IL-6, are able to induce inflammatory responses and aggravate the development of inflammation [28]. In response to LPS, pro-inflammatory cytokines, such as TNF-α and IL-6, are often overproduced. In the present study, we showed that pretreatment with CPT significantly reduced the secretion of pro-inflammatory mediators including TNF-α and IL-6 in LPS-stimulated RAW264.7 macrophages (Fig. 2a, b). Besides, by using flow cytometry assay, we also evaluated the effect of CPT on LPS-induced calcium influx. Our results demonstrated that CPT decreased calcium influx in LPS-stimulated RAW264.7 cells (Fig. 2c, d).

### CPT suppressed PI3K/AKT signaling pathway in LPS-stimulated RAW264.7 macrophages

**Fig. 3:**
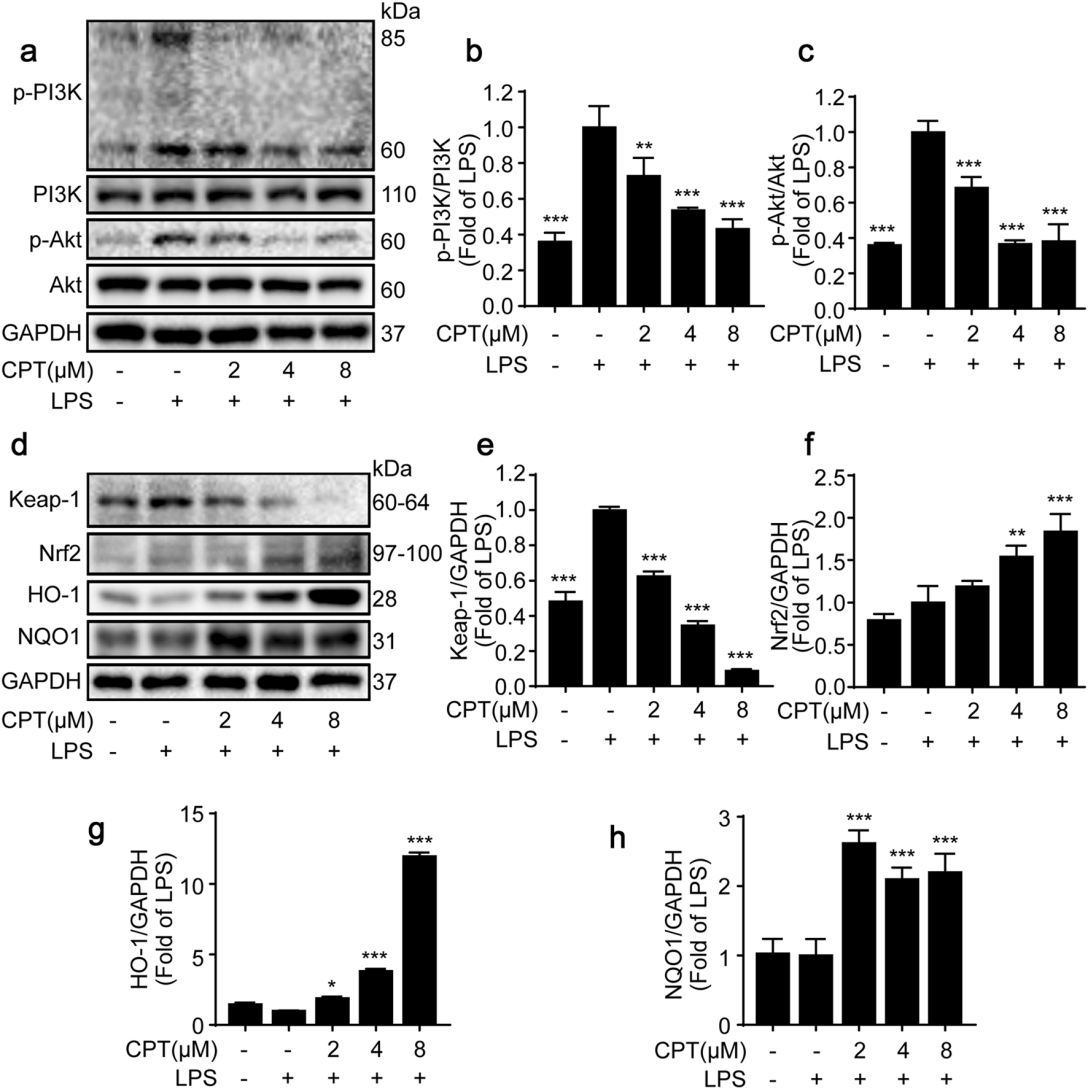
Effects of CPT on lipopolysaccharide (LPS)-stimulated phosphoinositide 3-kinase (PI3K)/AKT and Nuclear factor erythroid 2 related factor 2 (Nrf2) pathways in RAW264.7 cells. The cells were pretreated with CPT (0, 2, 4, 8 μM) for 1 h before exposure to LPS (1 μg/mL) for 8 h. The protein expressions of **a**, **b** PI3K, **a**, **c** AKT, **d**, **e** Kelch-like ECH-associated protein 1 (Keap-1), **d**, **f** Nrf2, **d**, **g** Heme oxygenase-1 (HO-1), and **d**, **h** NAD(P)H: quinone oxidoreductase-1 (NQO1) were determined by immunoblotting. n = 3. *p < 0.05, **p < 0.01, ***p < 0.001 compared with LPS alone group

The activation of PI3K/AKT signaling pathway plays a vital role in the progression of inflammation. PI3K is a family of enzymes involved in various cellular functions, and AKT is the effector of PI3K [10, 11, 13]. In order to investigate the effect of CPT on PI3K/AKT pathway, we measured the protein expressions of phosphorylated-PI3K and phosphorylated-AKT in LPS-induced RAW264.7 cells. Our results demonstrated that CPT suppressed the phosphorylation of both PI3K and AKT without altering the total PI3K and AKT protein expressions (Fig. 3a–c).

### CPT activated Nrf2 signaling pathway in LPS-stimulated RAW264.7 macrophages

A bulk of evidences has proved that Nrf2 signaling is involved in attenuating inflammation-associated pathogenesis [29, 30]. Nrf2 is one of the important nuclear transcription factors, which coordinately regulates constitutive and inducible expressions of anti-oxidant and phase 2 detoxification enzymes, such as NQO1 and HO-1 [17, 31–33]. The results demonstrated that CPT significantly increased Nrf2, HO-1 and NQO1 protein expressions, and markedly decreased Keap-1 protein expression in LPS-stimulated RAW264.7 cells (Fig. 3d–h).

### CPT inhibited NF-κB and MAPK pathways in LPS-stimulated RAW264.7 macrophages

**Fig. 4:**
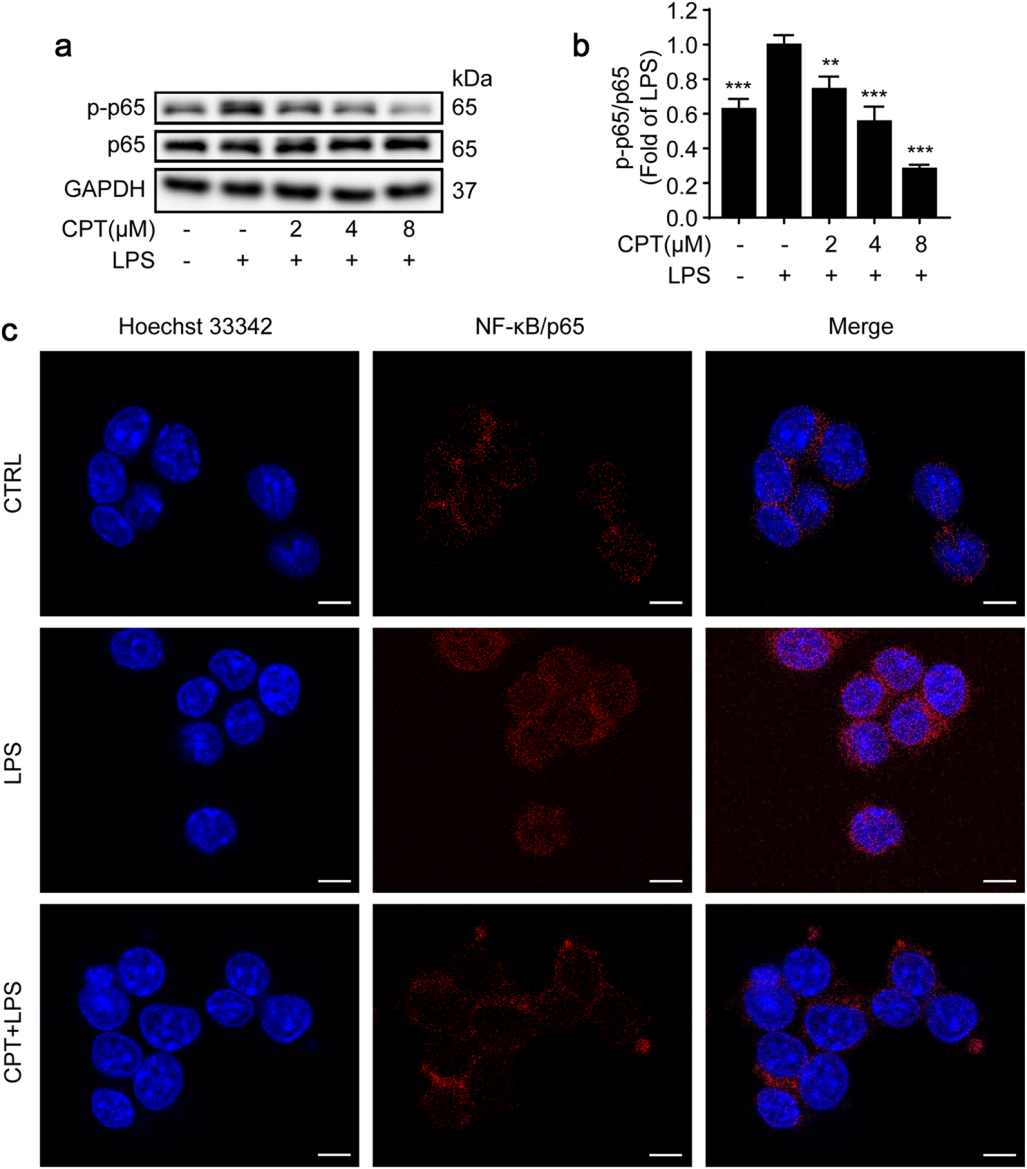
Inhibitory effects of CPT on lipopolysaccharide (LPS)-induced nuclear factor-kappa B (NF-κB)/p65 activation and nuclear translocation in RAW264.7 cells. **a**, **b** The cells were pretreated with CPT (0, 2, 4, 8 μM) for 1 h before exposure to LPS (1 μg/mL) for 8 h. The protein expression of p65 was determined by immunoblotting. n = 3. **c** The cells were pretreated with CPT (0, 2, 4, 8 μM) for 1 h before exposure to LPS (1 μg/mL) for 2 h. The nuclear translocation of NF-κB/p65 was detected by immunofluorescence. n = 3. **p < 0.01, ***p < 0.001 compared with LPS alone group. Scale bar: 5 μm

**Fig. 5:**
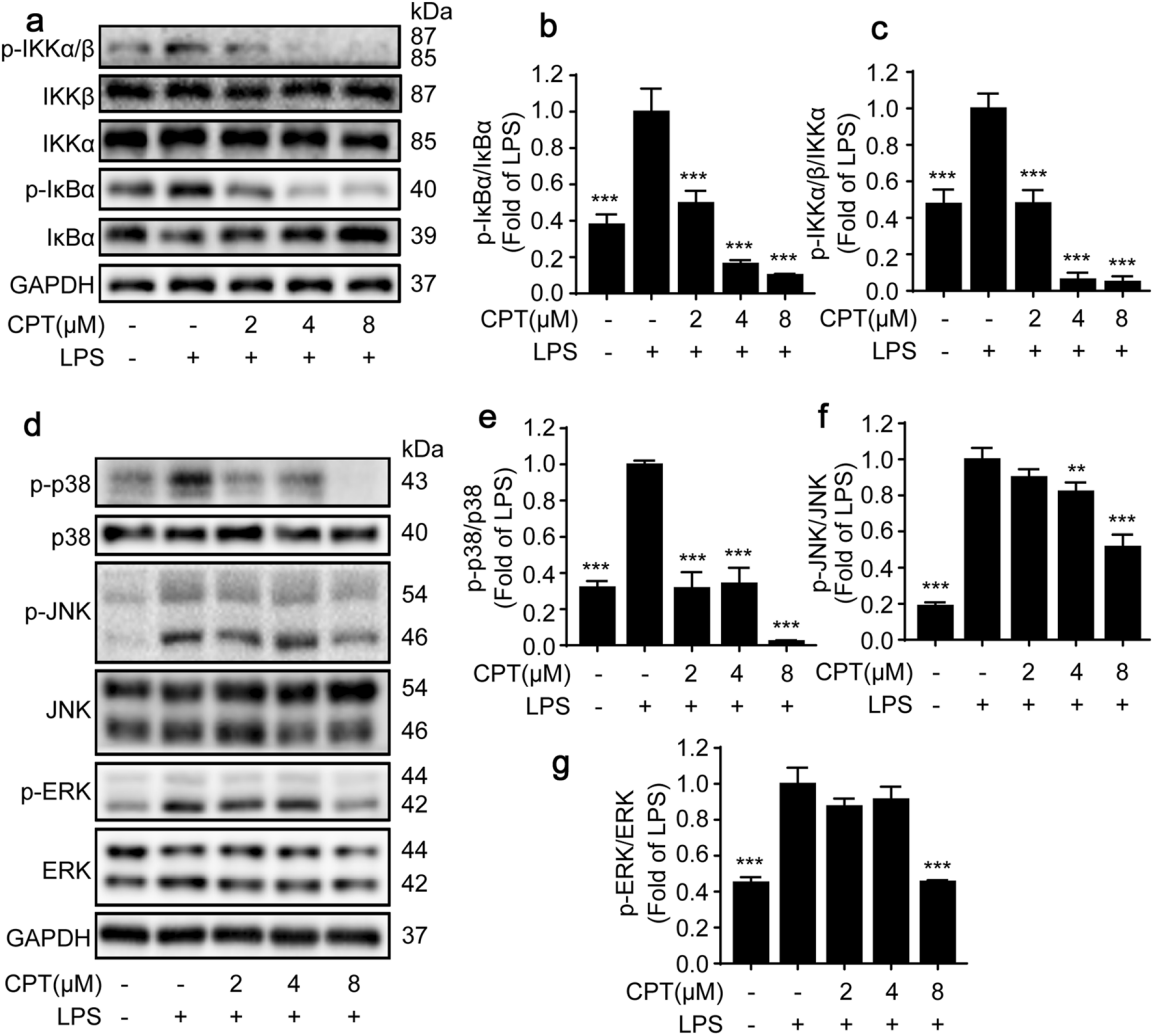
Inhibition of nuclear factor-kappa B (NF-κB) and mitogen-activated protein kinase (MAPK) pathways were participated in the anti-inflammatory process of CPT in RAW264.7 cells. The cells were pretreated with CPT (0, 2, 4, 8 μM) for 1 h before exposure to lipopolysaccharide (LPS; 1 μg/mL) for 8 h. The protein expressions of **a**, **b** inhibitor of kappa B α (IκBα), **a**, **c** inhibitor of kaapa B kinase (IKK)α/β, **d**, **e** p38, **d**, **f** c-Jun N-terminal kinase (JNK), **d**, **g** extracellular signal-regulated kinase (ERK) were determined by immunoblotting. n = 3. **p < 0.01, ***p < 0.001 compared with LPS alone group

Abundant studies demonstrated that the activation of NF-κB signaling pathway by LPS plays a significant role in the development and progression of inflammation [34, 35]. As shown in Fig. 4a, b, CPT significantly suppressed LPS-induced phosphorylation of p65 without altering the total expression of p65. Furthermore, the immunofluorescence results revealed that CPT dampened LPS induced translocation of NF-κB/p65 from the cytoplasm to the nucleus in RAW264.7 macrophages (Fig. 4c). In addition to NF-κB activity and translocation, CPT also exerted a strong effect on NF-κB pathway. The results showed that pretreatment of CPT inhibited IκB-α phosphorylation and degradation in LPS-stimulated RAW264.7 macrophages (Fig. 5a, b). In addition, we also determined the expressions of two upstream kinases of IκB. As shown in Fig. 5a, c, CPT pretreatment significantly suppressed IKK-α/β phosphorylation without altering the total IKK-α/β expression.

In addition to NF-κB pathway, MAPK pathway is another important mechanism contributing to the development of inflammation [2, 36]. The suppression of the phosphorylation of ERK1/2, JNK1/2, p38 in the MAPK pathway could effectively alleviate the occurrence of inflammatory diseases [35] Therefore, we investigated whether CPT could block the activation of MAPK cascades. As shown in Fig. 5d–g, CPT pretreatment significantly inhibited the phosphorylation of ERK1/2, JNK1/2 and p38 without affecting the total expressions of ERK1/2, JNK1/2, and p38. Collectively, we suggested that the suppression of NF-κB and MAPK pathways were participated in the anti-inflammatory effects of CPT.

### CPT exerted anti-inflammatory effects via inhibiting TLR4/MyD88 pathway in RAW264.7 macrophages

**Fig. 6:**
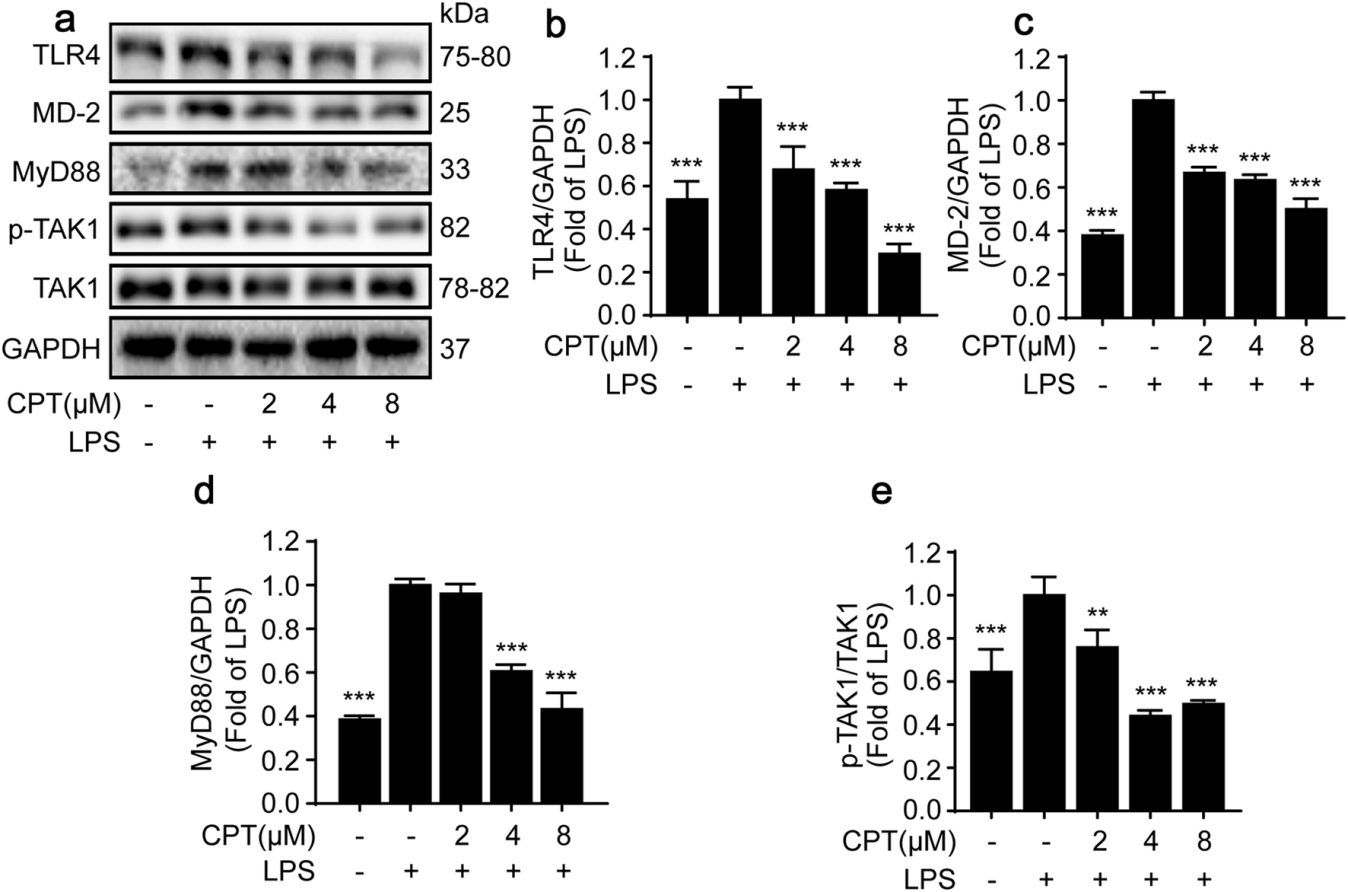
CPT exerted anti-inflammatory effects via toll-like receptor-4 (TLR4)/myeloid differentiation factor 88 (MyD88) pathway in RAW264.7 cells. The cells were pretreated with CPT (0, 2, 4, 8 μM) for 1 h before exposure to lipopolysaccharide (LPS; 1 μg/mL) for 8 h. The protein expressions of **a**, **b** TLR4, **a**, **c** MD-2, **a**, **d** MyD88, and **a**, **e** transforming growth factor beta-activated kinae 1 (Tak1) were determined by immunoblotting. n = 3. **p < 0.01, ***p < 0.001 compared with LPS alone group

When LPS is released from the bacterial membrane, it will be sensed by the TLR4–MD-2 heterodimer [4, 5]. The TLR4 heterodimer will then recruit the downstream protein MyD88, which is often needed to activate NF-κB and MAPK pathways during inflammatory processes [5, 7]. To explore the effect of CPT on TLR4/MyD88 pathway, we measured the protein expressions of TLR4, MD-2, and MyD88. As shown in Fig. 6a–d, LPS markedly up-regulated the protein expressions of TLR4, MD-2, and MyD88, and these were suppressed by CPT. Furthermore, the protein expression of Tak1, a downstream signal protein of TLR4-MyD88, was also determined. Our data showed that CPT significantly suppressed Tak1 phosphorylation without affecting the total Tak1 expression in LPS-stimulated RAW264.7 cells (Fig. 6a, e).

## Discussion

Inflammation, a complicated biological process triggered by harmful inducers such as pathogens or irritants, is characterized by the secretion of pro-inflammatory mediators such as TNF-α, IL-6, IL-1β, NO, and prostaglandin E_2_ (PGE_2_) [35, 37]. Therefore, blockade of the secretion of these inflammatory mediators could be a highly effective strategy for alleviating the development and progression of inflammatory diseases. Danshen, a traditional Chinese medicine, has been widely used to treat inflammation-related diseases [38]. In China, many drugs that involve Danshen, such as Fufang Danshen drop pills (Z10950111) and tanshinones capsules (Z13020110), have already been approved by the China Food and Drug Administration. CPT, a major tanshinone of Danshen, exhibited a wide range of bioactivities, especially anti-inflammatory activity. Although the anti-inflammatory effects of CPT were demonstrated before [23, 24], the detailed underlying mechanisms have not been clearly understood. In the present study, we investigated the anti-inflammatory effects of CPT and its underlying mechanisms in mouse macrophages, RAW264.7 cells.

In this study, we showed that 2–8 μM CPT exhibited no toxicity to RAW264.7 macrophages. A study reported that CPT at a dosage of 100 μM exhibited no cytotoxicity [20]. However, we found that CPT at a dosage of 10 μM already displayed significant cytotoxicity in RAW264.7 macrophages (data not shown). Therefore, the maximum concentration of CPT that we used in our study was 8 μM. Moreover, our data showed that CPT effectively inhibited LPS-induced secretion of NO, TNF-α and IL-6 in RAW264.7 macrophages. We also demonstrated that CPT down-regulated the key inflammatory proteins, iNOS and COX-2 in a concentration-dependent manner. This suggested that CPT attenuated inflammatory responses in RAW264.7 macrophages. This was consistent with previous studies, showing that CPT possessed anti-inflammatory effects [23, 24]. However, a study showed that the anti-inflammatory effects of CPT were directly through inhibiting COX-2 enzyme activity in insect sf-9 cells, but not the transcription or translation of this enzyme [39]. This inconsistent result was probably due to different species used. Furthermore, an increase in intracellular calcium was shown to activate NF-κB, which could regulate the transcription of pro-inflammatory cytokines [40]. Previous study indicated that that the rise of intracellular Ca^2+^, through opening of L-voltage-sensitive Ca^2+^ channels (L-VSCCs) at the plasma membrane and indirect opening of In3P receptors associated with the intracellular stores of Ca^2+^, is responsible for the basal NF-κB activity [41]. In addition, Ca^2+^ influx affects NF-κB at the level of both nuclear translocation and transcriptional activity. Thus, suppression of calcium influx decreases phosphorylation of NF-κB proteins and translocation from the cytoplasm into the nucleus, suggesting that the results of calcium are in line with the NF-κB signalling pathway [41]. Our results showed that CPT significantly decreased intracellular calcium levels, suggesting that the anti-inflammatory effects of CPT could be via inhibition of calcium influx. TLR4, one of the best characterized pattern recognition receptors, binds to LPS from Gram-negative bacteria [42, 43], and TLR4 and MD-2 form a heterodimer [44]. When LPS is sensed by TLR4, TLR4 heterodimer will recruit MyD88 and/or TIR-domain-containing adapter-inducing interferon-β (TRIF) [42]. MyD88, the downstream protein of TLR4, is often needed to activate TAK1. Subsequently, NF-κB and MAPK pathways are activated during inflammatory processes [4, 42]. Our results showed that CPT reduced the protein expressions of TLR4 and MD2 in LPS-stimulated macrophages. In addition, CPT significantly inhibited the phosphorylation of IKKα/β, IκBα, and NF-κB/p65, as well as NF-κB/p65 nuclear translocation in LPS-stimulated macrophages. These results indicated that inhibition of NF-κB signaling was involved in the anti-inflammatory effects of CPT. In addition, MAPK pathway (JNK1/2, ERK1/2 and p38MAPK) can also regulate inflammatory responses and immune defense [45]. Our results demonstrated that CPT pretreatment could dramatically attenuate the phosphorylation of ERK, JNK and p38 in LPS-stimulated RAW264.7 macrophages. It suggested that CPT suppressed NF-κB and MAPK signaling pathways to exert anti-inflammatory effects in LPS-stimulated RAW264.7 macrophages. This was consistent with previous study, showing that MAPK pathway could be regulated by CPT [46]. Similarly, a recent study reported that CPT exerted anti-inflammatory effects via MAPK and NF-κB pathways [46]. Furthermore, PI3K/Akt signaling can also be activated by LPS-induced TLR4 pathway, and it has been demonstrated to play a critical role in inflammatory responses [47]. AKT and PI3K are the key proteins involved in PI3K/AKT pathway. Therefore, we have also studied the effects of CPT on PI3K/AKT signaling pathway in RAW264.7 macrophages. We found that CPT could significantly inhibit the phosphorylation of PI3K and AKT. This suggested that CPT suppressed PI3K/AKT pathway to exert its anti-inflammatory effects in LPS-induced macrophages. Similarly, CPT was also shown to mediate anti-inflammatory effects via inhibition of PI3K/AKT pathway in a rat model of neuropathic pain [24]. In addition, previous study indicated that there is a crosstalk between PI3K/AKT and MAPK in cellular protection and proliferation. Paul illustrated that signaling by the ERK1/2 pathway can also stimulate release of growth factors which can feed back onto tumor cells to re-energize signaling pathways [48, 49]. Specifically, PI3K-AKT and MAPK-ERK1/2 pathways in a cell type-dependent fashion can collaborate to maintain cell viability [49].

Nrf2 does not only regulate oxidative stress, but also represses inflammation by regulating cytokine secretion [13, 15]. In the present study, we also determined the involvement of Nrf2 pathway in mediating the anti-inflammatory effects of CPT in LPS-induced RAW264.7 cells. We showed that CPT pretreatment could markedly up-regulate NQO1 and HO-1, as well as its upstream Nrf2. Moreover, the expression of Keap-1 protein was suppressed after CPT pretreatment in LPS-induced macrophages. This indicated that CPT could inhibit oxidative stress by activating Nrf2 signaling pathway in RAW264.7 macrophages. This was consistent with another study, showing that CPT could activate Nrf2/HO-1 pathway in BV-2 microglial cells [23].

## Conclusions

**Fig. 7:**
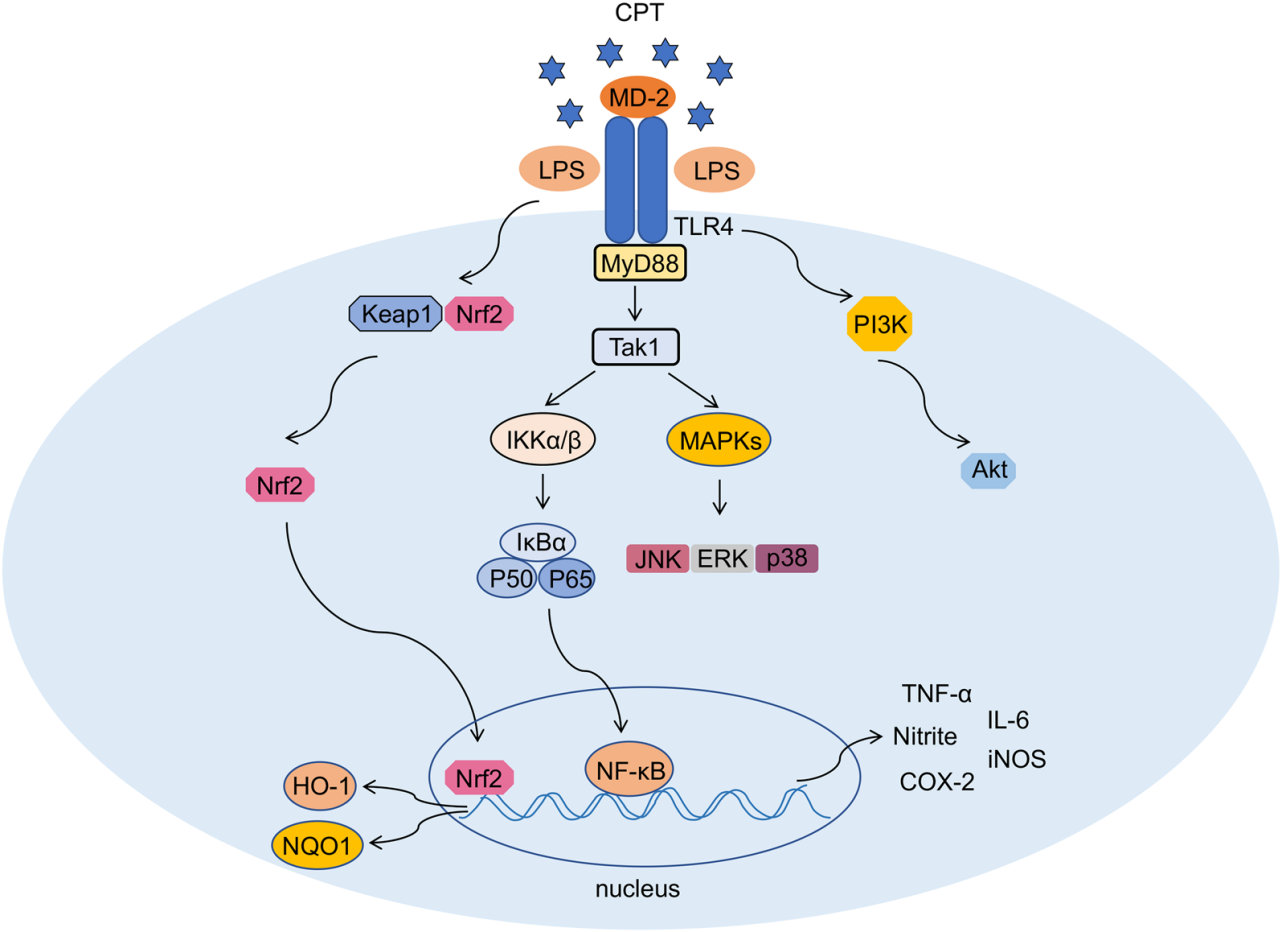
The schematic of CPT’s anti-inflammatory mechanism in LPS-stimulated RAW264.7 cells

Our findings demonstrated that CPT possessed anti-inflammatory effects in LPS-stimulated RAW264.7 macrophages. We showed that it exerted anti-inflammatory effects through multiple pathways. The underlying mechanisms were through the inhibition of PI3K/AKT, NF-κB and MAPK signaling pathways, and activation of Nrf2 pathway (Fig. 7). Taken together, we suggested that CPT has beneficial effects on alleviating inflammatory responses in mouse macrophages, RAW264.7, and could be a potential drug for the treatment of inflammatory diseases.

## Data Availability

Not applicable.

## Abbreviations

COX-2: Cyclooxygenase-2
CPT: Cryptotanshinone
DAPI: 4ʹ,6-Diamidino-2-phenylindole
DMEM: Dulbecco’s modified eagle medium
DMSO: Dimethyl sulfoxide
ELISA: Enzyme-linked immunosorbent assay
ERK: Extracellular signal-regulated kinase
FBS: Fetal bovine serum
HO-1: Heme oxygenase-1
HRP: Horseradish peroxidase
IL: Interleukin
IκBα: Inhibitor of kappa B α
IKK: Inhibitor of kappa B kinase
iNOS: Inducible nitric oxide synthase
JNK: c-Jun N-terminal kinase
Keap-1: Kelch-like ECH-associated protein 1
LPS: Lipopolysaccharide
MAPK: Mitogen-activated protein kinase
MTT: 3-(4,5-dimethylthiazol-2-yl)-2,5-diphenyl tetrazolium bromide
MyD88: Myeloid differentiation factor 88
NF-κB: Nuclear factor-kappa B
NO: Nitric oxide
Nrf2: Nuclear factor erythroid 2 related factor 2
NQO1: NAD(P)H: quinone oxidoreductase-1
PGE_2_: Prostaglandin E_2_
PI3K: Phosphatidylinositol 3-kinase
PVDF: Polyvinylidene fluoride
SD: Standard deviation
Tak1: Transforming growth factor beta-activated kinase 1
TLR4: Toll-like receptor-4
TNF: Tumor necrosis factor
TRIF: TIR-domain-containing adapter-inducing interferon-β
